# Initial *In Vivo* Quantification of Tc-99m Sestamibi Uptake as a Function of Tissue Type in Healthy Breasts Using Dedicated Breast SPECT-CT

**DOI:** 10.1155/2012/146943

**Published:** 2012-08-16

**Authors:** Steve D. Mann, Kristy L. Perez, Emily K. E. McCracken, Jainil P. Shah, Terence Z. Wong, Martin P. Tornai

**Affiliations:** ^1^Medical Physics Graduate Program, Duke University, Durham, NC 27710, USA; ^2^Department of Radiology, Duke University Medical Center, Durham, NC 27710, USA; ^3^Duke University Medical School, Durham, NC 27710, USA; ^4^Department of Biomedical Engineering, Duke University, Durham, NC 27710, USA

## Abstract

A pilot study is underway to quantify *in vivo* the uptake and distribution of Tc-99m Sestamibi in subjects without previous history of breast cancer using a dedicated SPECT-CT breast imaging system. Subjects undergoing diagnostic parathyroid imaging studies were consented and imaged as part of this IRB-approved breast imaging study. For each of the seven subjects, one randomly selected breast was imaged prone-pendant using the dedicated, compact breast SPECT-CT system underneath the shielded patient support. Iteratively reconstructed and attenuation and/or scatter corrected images were coregistered; CT images were segmented into glandular and fatty tissue by three different methods; the average concentration of Sestamibi was determined from the SPECT data using the CT-based segmentation and previously established quantification techniques. Very minor differences between the segmentation methods were observed, and the results indicate an average image-based *in vivo* Sestamibi concentration of 0.10 ± 0.16 **μ**Ci/mL with no preferential uptake by glandular or fatty tissues.

## 1. Introduction

Mammography is considered the gold standard for the detection and prebiopsy diagnosis of breast cancer; however, other imaging modalities are beginning to emerge that may allow for alternative techniques for diagnosis or staging of tumors. One such technique developed in our lab is the use of a dedicated breast single photon emission computed tomography and X-ray computed tomography (SPECT-CT) system. Digital mammography suffers from the overlapping of tissues, resulting in difficulty interpreting images in patients with dense breasts. Fully 3-dimensional (3D) imaging modalities, such as CT, are able to overcome the problems associated with limited-view planar systems of mammography or pseudo-3D tomosynthesis. In addition, the diagnostic SPECT system offers unique functional molecular information about the tissue, which may allow for more accurate diagnostics, staging, and treatment response of malignant tissue. Currently, commercially available nuclear medicine breast imaging systems (breast specific gamma imaging or molecular breast imaging systems) utilize planar detectors with compression and limited angular views based on repositioned camera similar to traditional X-ray mammography [1]. Although these systems offer useful information not attainable with conventional mammography and often yield otherwise occult lesions, especially in dense breasted women, the 2-dimensional nature of the systems intrinsically limits their ability to quantify the uptake of radiotracers, such as Tc-99m Sestamibi (MIBI) [2–4]. Additionally, the use of compression adds to the discomfort and apprehension of patients to have such scans. While breast MRI is also compression-free, it is not without discomforts, with common problems including that some women are unable to altogether enter the gantry, the need for sedatives to help others [5], and universally noted chest (sternal) pain from positioning even though their breasts are not compressed. Additionally, pacemakers and other electromagnetic susceptible devices may complicate MRI procedures. Our system eliminates many of the issues by having a comfortable patient support to rest on and allowing the breast to remain uncompressed while obtaining fully 3D images using a dedicated breast SPECT-CT system located underneath the patient [6].

Tc-99m Sestamibi (MIBI) is a common nuclear medicine imaging agent which has shown to have preferential uptake in breast cancer tissue, with an average reported uptake of 6 : 1 compared to background [7]. However, *in vivo *quantifiable results for MIBI tracer uptake in normal, healthy tissue have yet to be determined. By measuring the average baseline uptake of MIBI in breast tissue, we hypothesize that it may be possible to establish a global threshold for improved diagnosis and staging, especially in women with dense breasts. The objective of this initial study is to measure the average uptake of MIBI in women without any breast cancer history and to determine any differences in MIBI uptake in glandular and fatty breast tissue through the use of various segmentation techniques of the CT images.

## 2. Materials and Methods

Our dedicated breast SPECT-CT system is described extensively in the literature [6, 8–10]. The CT subsystem consists of a digital 25 × 20 cm^2^ flat-panel detector (Paxscan 2520, *Varian Medical Systems, Inc*.) having 127 micron pixelation, and RAD94 (*Varian Medical Systems, Inc*.) tungsten target X-ray source with a cerium-filtered quasimonochromatic X-ray beam with mean energy 35 keV. The SPECT subsystem consists of a 16 × 20 cm^2^ cadmium-zinc-telluride (CZT) LumaGEM 3200S (*Gamma Medica, Inc*.) gamma camera with 2.5 mm pixelation for SPECT imaging mounted orthogonally to the CT subsystem; the SPECT subsystem is capable of fully breast-contoured, sinusoidal trajectories, which allow improved imaging of the chest wall and axilla. The CZT camera has an excellent energy sensitivity and measured resolution of 6.7% at 140 keV, the emission energy of Tc-99m-MIBI. While the CT sub-system is limited to azimuthal rotation, the CZT camera is capable of three degrees of motion: azimuthal rotation, polar angle rotation, and radial position. The numerous motion stages allow for breast-specific contoured trajectories for maximum resolution and imaging volume.

**Figure 1:**
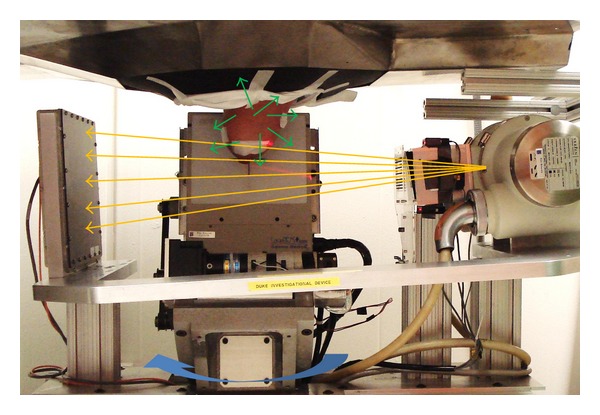
Photograph of the dedicated breast SPECT-CT system. The CT subsystem (left and right) emits a quasimonochromatic X-ray cone beam (orange arrows) with 35 keV mean energy. A rotating plate in front of the CT collimator allows for scatter projection acquisitions. Ribbon lasers mounted on the collimator allow for rapid positioning of the breast into the common FOV. The CZT gamma camera (behind breast) used for SPECT imaging is mounted orthogonally to the CT subsystem and passively detects 140 keV gammas (green arrows) from MIBI.

As part of an approved IRB study, seven subjects undergoing presurgical diagnostic parathyroid imaging studies were consented for imaging using our SPECT-CT system. A library database search reveals no link between hyperparathyroidism (one of the most common reasons for parathyroid nuclear imaging at Duke) and breast cancer, save some case reports in the 1970s [11], making this normal-risk population ideal for our studies, where we additionally considered lowering the radiation risk from radiochemical injections to otherwise healthy volunteers. Subjects were scanned in between their routine scintigraphy (10 min post 25 mCi injection) and SPECT (2 hrs post injection) diagnostic parathyroid scans. Subjects' age (34–64), weight, and menstrual cycle were recorded. Volunteers were marked using dual-modality fiducial markers and asked to lie down on a custom radioopaque bed with a flexible center region and opening, allowing one pendant breast to suspend freely in the common field of view (FOV) of the dual-modality system (Figure 1). First, 240 CT cone beam projections using stop-and-shoot were collected in a clockwise fashion about 360°; then, 6 equally distributed scatter projection measurements using a beam stop array (BSA) were acquired in a counterclockwise fashion and subsequently used to scatter correct the CT projections [12]. The total CT scan time was approximately 8 minutes. Finally, 128 SPECT projections (5 s per projection, ~12 min total) using a contoured projected sine wave (PROJSINE) trajectory were acquired [6]. The SPECT data were acquired in list mode and resorted to an 8% primary photopeak energy window about the 140 keV peak of Tc-99m and ~30% scatter window below and abutting the photopeak window. Using quantification procedures previously evaluated on phantoms, the SPECT data were scatter corrected using the dual-energy window method, and attenuation corrected using a SPECT-based uniform attenuation mask with attenuation coefficient of water at 140 keV (0.1545 cm^−1^) [6]. Resultant images were decay corrected to the time of injection for analysis. This procedure yields an *in vivo *quantifiable SPECT image volume. To determine the average MIBI concentration in glandular and fatty tissue, specific volumes of interest need to be generated using the segmented CT data, described in the following.

**Figure 2:**
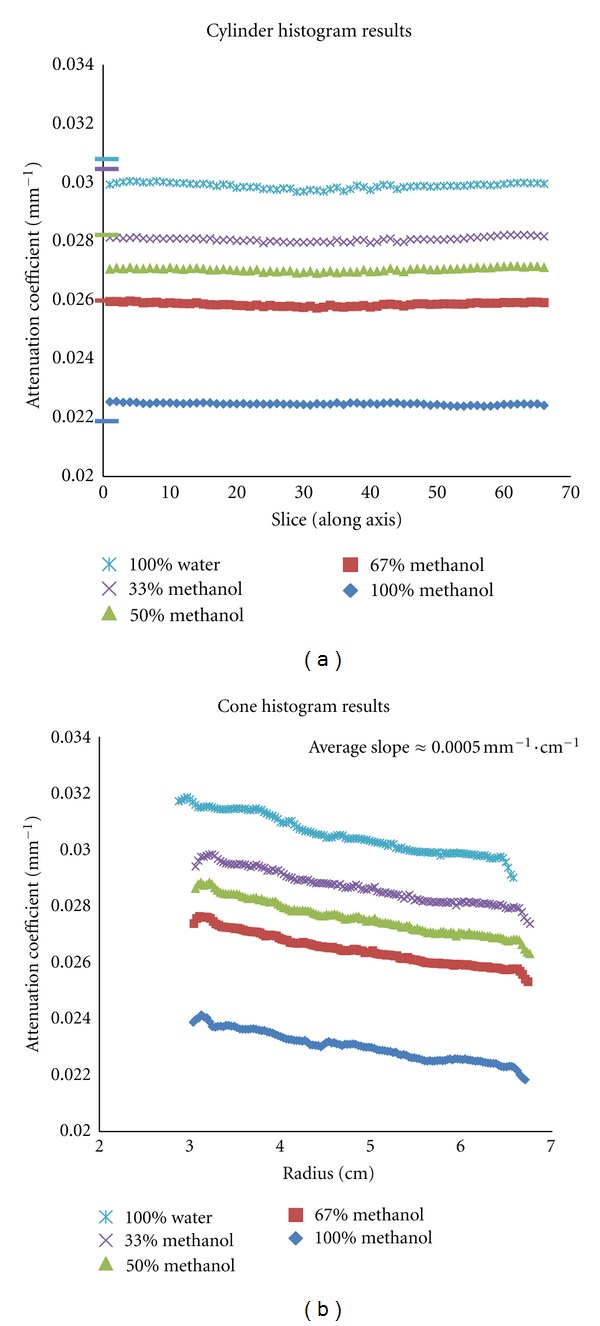
Plots of the centroids of the fitted Gaussian functions for BSA scatter-corrected (a) cylinder and (b) cone phantoms using various mixtures of methanol and water to simulate different uniform tissue densities. NIST-based attenuation values are indicated by matching tick marks on the *y*-axis of the cylinder graph. The cone phantom results indicate a significant dependence of the measured attenuation value on object size.

**Figure 3:**
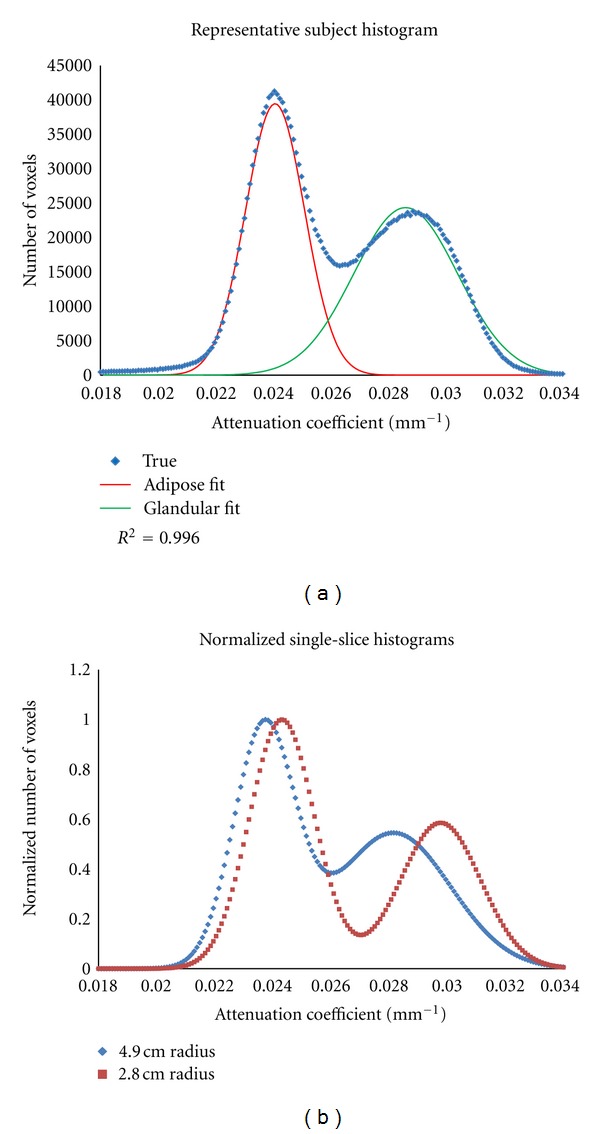
(a) The graph illustrates a representative subject's breast volume histogram and corresponding dual-Gaussian fit. The *R* ^2^ value indicates a high quality fit, allowing for various segmentation methods based on the minimum, cross-point, and percentage contribution to be compared. (b) Two normalized representative single-slice histograms from the same subject, along with estimated breast size. The graph clearly demonstrates the shift in attenuation coefficients as a function of object radius.

The reconstructed scatter-corrected CT data were analyzed and segmented according to attenuation coefficients. To validate the procedure, varying diameter (6–14 cm) cone and 12.5 cm diameter cylinder phantoms filled with varying mixtures of water and methanol (100% water to 100% methanol in uniform intervals, to simulate breast tissue) were imaged [13], scatter corrected using our BSA correction technique, and analyzed to determine any postscatter correction effects of object size on the histogram of attenuation values in the image volume (see ahead to Figure 3). The histograms of each slice of the cylinders and cones were fitted to a Gaussian function using nonlinear least squares methods, and the centroids were plotted as a function of cone radius. The results, shown in Figure 2, demonstrate that regardless of the slice position in the cylinder, there is no effect of the intersection of the cone beam X-ray source and fixed diameter of the uniformly filled cylinder; the only effect in attenuation value difference is that due to the nature of the material. The differences between measured and NIST-based narrow beam attenuation coefficients are minor, with the importance being the robustness of the measured value from the cylinder data. A significant dependence of the centroids on object size is noted, however, which may adversely affect simple threshold methods for segmentation.

For the study subjects' data, an edge detection algorithm was used to automatically remove the skin boundary of the breast from the scatter-corrected reconstructed CT images. For comparison, three segmentation techniques of varying computational levels were used for volumetric separation of the glandular and adipose breast tissue. Histograms of reconstructed image slices were first fit with a dual-Gaussian function, defined in (1):

(1) A1e−(x−B1)2/C12+A2e−(x−B2)2/C22.

The simplest method used the entire breast histogram for the curve fitting, with a hard threshold at the minimum between the two Gaussians (Figure 3) used to segment fatty and glandular image-based tissue types. However, this method has potential problems due to the observed radial dependence of the reconstructed attenuation numbers, as described above. This object-size dependence may lead to incorrect binning of pixels when using a global threshold for segmenting a breast. Thus, two additional segmentation techniques were developed to analyze the data on a slice-by-slice basis accounting for the object size.

**Figure 4:**
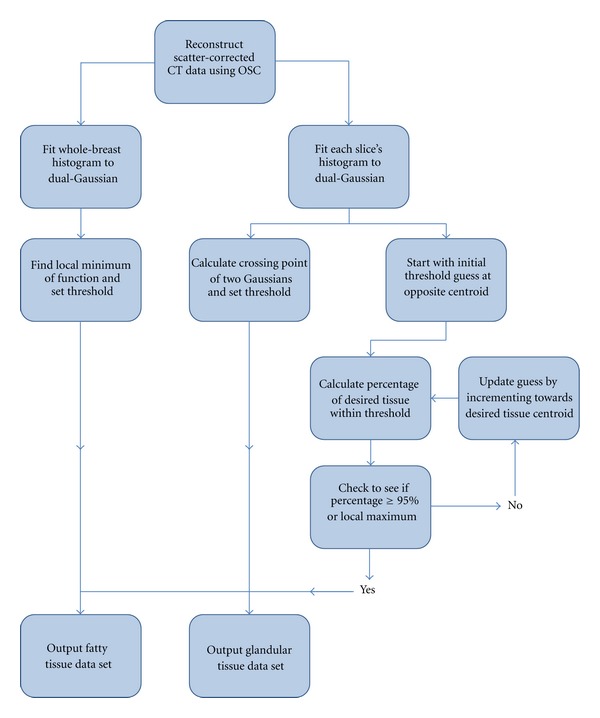
Flowchart diagraming the three segmentation procedures. Each of the three methods uses a dual-Gaussian function to fit the histograms, but the method for choosing an appropriate threshold varies. The simplest method involves choosing the minimum of the total histogram, while more complex methods involve analyzing the percent contribution of each tissue type within the threshold bounds.

Both alternative methods utilized the same dual-Gaussian fitting procedure, but for each individual slice (Figure 4). For the first method, the crossing point between the centroids of the two Gaussian functions was calculated and used as a threshold to segment each slice. Depending on the area of the primary image-based breast tissue components, this crossing point was located to either side of the visible minimum of the dual-Gaussian fit. The second method used an iterative approach to find threshold values for each slice that would result in images consisting of at least 95% of the desired tissue type; for slices where the Gaussian overlap was significant, the maximum achievable percentage was chosen.

After segmentation, all nonsegmented CT data sets were coregistered to the SPECT data using the fiducial markers and AMIDE software. Next, MIBI concentrations were measured for the whole breast as well as fatty and glandular tissues from the volumes of interest derived from the three described segmentation procedures. Images were also given to radiologists to read for additional confirmation of our cancer-free assumption.

## 3. Results

**Figure 5:**
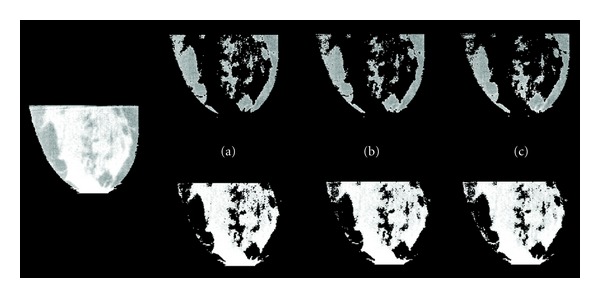
Slice through a representative subject's breast, with the skin removed (left). Segmented images (right) from the same breast using (a) minimum, (b) cross-point, and (c) 95% interval methods described in the text. All images are equivalently windowed and leveled. The minor differences in the resultant images due to the segmentation methods may indicate a lack of need for procedures more sophisticated than a simple threshold.

Figure 5 shows the results of the different segmentation methods for one representative data set. Note that the most anterior (nipple) and posterior (chest wall) breast regions were truncated to avoid the reconstruction artifacts in those regions.

The results of the quantitative *in vivo* MIBI measurements are given in Table 1 for each of the three segmentation procedures. Results indicate a lack of tissue-specific preferential MIBI uptake, with an overall average (for the seven subjects) uptake value of 0.10 ± 0.16 *μ*Ci/mL for the whole breast. Three of the seven subjects moved significantly during the CT scan or otherwise had truncation artifacts due to the size of the breast; those subjects were excluded from the detailed segmentation analysis.

## 4. Discussion

Overall *in vivo* quantification results in this initial study indicate an average activity concentration of 0.10 ± 0.16 *μ*Ci/mL, but with a fairly large variance across all patients. The large variance is largely due to the low-count statistics obtained by SPECT due to acquisition time restrictions in a clinical setting, with the majority of voxels having a measured uptake of 0 *μ*Ci/mL. We have previously measured the overall sensitivity of the SPECT system to be accurate for a variety of breast phantom sizes and activity concentrations down to 0.03 *μ*Ci/mL [6]. The mean results fall within the expected range, as compared with the literature, when accounting for the various corrections applied to our data [14]. Additional subjects may help reduce the variance and allow greater confidence in the expected average MIBI uptake. Results further indicate no preferential uptake of MIBI in glandular or fatty breast tissue. They are also independent of the segmentation method used; this is likely due to the poorer resolution of SPECT (2.5 mm voxels) compared with CT (0.508 mm voxels), which yields minimal gains in accuracy for improved segmentation and registration procedures.

The lack of preferential uptake with MIBI differs from results seen using FDG-PET breast cancer imaging based on clinical scans, likely due to metabolic differences of the tracers [15], but also potentially due to the higher resolution results possible with dedicated imaging modalities. That is, the FDG-PET uptake has not yet been quantified with higher resolution breast PET imaging modalities. Furthermore, our dedicated SPECT system has an intrinsic resolution of 2.5 mm, while clinical SPECT scanners have intrinsic resolutions about 7 mm, before the additional resolution degradation with object distance is considered. Our system has routinely yielded visibility of objects greater than or equal to 4 mm diameters [16] and with accurate quantification.

Approximately 30 subjects are necessary to achieve a 95% confidence in total breast quantification results. Further study on breast cancer confirmed subjects (BIRADS 4 or 5 subjects scheduled for biopsy) may provide complementary information on MIBI uptake concentrations of both the patients' foci as well as the assumed “normal” surrounding tissue. Such results may allow for the development of a universal threshold for diagnosing early-stage breast cancer or be used in staging breast cancer as well as monitoring therapeutic response.

## 5. Conclusions

No tissue-specific distinction in MIBI uptake was seen in this initial study between glandular and adipose tissues. This implies that menstrual cycle or other biological factors may not affect routine breast imaging with MIBI with respect to baseline background uptake and distribution. One caveat with our results, however, is that more patient data is needed for higher confidence in these initial results. The results are independent of three implemented segmentation procedures, indicating that a simple hard-threshold method may be sufficient for subsequent segmentation of corrected, dedicated breast CT-imaged breast tissue. The potential to use a global lower-level threshold in SPECT to identify regions of interest, especially within dense breasts where we have not observed any detrimental effects, may allow for improved patient care. Further study on normal, otherwise healthy patients is necessary to provide greater confidence in the expected mean MIBI uptake.

## Figures and Tables

**Table 1:** Results tabulated from the seven patients show no preferential uptake of MIBI in glandular or fatty breast tissue. Average measured uptake values are noisy due to count limitations of *in vivo *SPECT measurements.

Subject	Total breast	Adipose tissue			Glandular tissue		
		Minimum	Cross-point	95% interval	Minimum	Cross-point	95% interval
	Mean (*μ*Ci/mL)	Mean (*μ*Ci/mL)	Mean (*μ*Ci/mL)	Mean (*μ*Ci/mL)	Mean (*μ*Ci/mL)	Mean (*μ*Ci/mL)	Mean (*μ*Ci/mL)
1	0.176 ± 0.486	0.154 ± 0.624	0.155 ± 0.623	0.158 ± 0.624	0.20 ± 0.753	0.196 ± 0.747	0.196 ± 0.745
2	0.061 ± 0.388	0.055 ± 0.458	0.054 ± 0.451	0.053 ± 0.471	0.09 ± 0.579	0.082 ± 0.555	0.084 ± 0.552
3	0.168 ± 0.506	0.175 ± 0.601	0.176 ± 0.601	0.179 ± 0.611	0.120 ± 0.492	0.139 ± 0.547	0.129 ± 0.504
4	0.069 ± 0.299	0.066 ± 0.366	0.065 ± 0.360	0.063 ± 0.347	0.077 ± 0.380	0.078 ± 0.394	0.079 ± 0.394
5	0.117 ± 0.520	—	—	—	—	—	—
6	0.038 ± 0.206	—	—	—	—	—	—
7	0.064 ± 0.385	—	—	—	—	—	—

Total	0.10 ± 0.16	0.11 ± 0.26	0.11 ± 0.26	0.11 ± 0.26	0.12 ± 0.27	0.12 ± 0.27	0.12 ± 0.27
